# Fast anodization fabrication of AAO and barrier perforation process on ITO glass

**DOI:** 10.1186/1556-276X-9-159

**Published:** 2014-04-03

**Authors:** Sida Liu, Zuzhou Xiong, Changqing Zhu, Ma Li, Maojun Zheng, Wenzhong Shen

**Affiliations:** 1 Key Laboratory of Artificial Structures and Quantum Control (Ministry of Education), Department of Physics and Astronomy, Shanghai Jiao Tong University, 800 DongChuan Road, 200240 Shanghai, China; 2 School of Chemistry & Chemical Technology, Shanghai Jiao Tong University, 800 DongChuan Road, 200240 Shanghai, China

**Keywords:** Fast anodization, AAO, Barrier layer, ITO glass

## Abstract

Thin films of porous anodic aluminum oxide (AAO) on tin-doped indium oxide (ITO) substrates were fabricated through evaporation of a 1,000- to 2,000-nm-thick Al, followed by anodization with different durations, electrolytes, and pore widening. A faster method to obtain AAO on ITO substrates has been developed, which with 2.5 vol.% phosphoric acid at a voltage of 195 V at 269 K. It was found that the height of AAO films increased initially and then decreased with the increase of the anodizing time. Especially, the barrier layers can be removed by extending the anodizing duration, which is very useful for obtaining perforation AAO and will broaden the application of AAO on ITO substrates.

## Background

Nanostructures with monodisperse arrangement nanopores have been used widely as template to fabricate various functional nanomaterials [1-4]. One of such nanostructures is well-known porous anodic aluminum oxide (AAO), which is considered as one of the most prominent template owing to its advantages of controllable diameter, high aspect ratio, and economical way in producing [1,5-7]. To this day, a variety of synthetic methods have been developed to fabricate porous AAO, typically fabricated from anodizing bulk aluminum foils or plates at constant voltage or current density in various electrolytes such as sulfuric redacid, oxalic acid, phosphoric acid, etc [8-11]. However, it needs great care in the process of preparation of the aluminum substrate and the manipulation of the anodic film since the AAO is a brittle ceramic film grown on soft aluminum metal [12]. Thus, direct fabricating AAO onto rigid substrates become a more convenient and important technique to prepare vertical nanostructures. The fabrication of AAO on Si substrates has been well established [12-17], while many photonic applications call for nanowire structures on transparent conductive substrates. The tin-doped indium oxide (ITO) glass is a good choice to satisfy this demand [18-20].

Recently, several articles have reported the fabrication and application of AAO in phosphoric acid [21-23]. Chu et al. [23] reported the successful fabrication of AAO is in phosphoric acid, from 2-µm thick aluminum films deposited by radio frequency (rf) sputtering, resulting in large-diameter AAO pores. An anodization duration of more than 40 min was observed in 10 vol.% phosphoric acid at a voltage of 130 V at 280 K. Small transverse holes appear regularly in the anodized films, which arose from the fact that the aluminum was deposited in two-step sputtering. The current density rapidly decreased to 0, indicating a loss of electrical conductivity. Moreover, the barrier layer still exists, preventing the physical and electrical contact between the pore and the substrate.

The barrier layer of AAO arouse many people’s attention since it makes the bottom of the AAO electrically isolated from the substrate. The method to get rid of the barrier layer has been proved to be the key to make electrical contact at the bottom. A current technology that removes the barrier layer is through immersion in dilute acid during which time the pores are also widened [12,24-26]. Oh et al. [22] had an innovative method through selectively etching the penetrating metal oxide WO_3_, which was formed from the metal underlayer W, to open the base of the alumina pores. However, it calls for a more simple method to remove the barrier layer.

In this article, fast growth of the AAO film on ITO glass was successfully realized by employing high-field anodization technology of our group [10] and a distinct ‘Y’ branch morphology was observed. The evolution process of the AAO film on ITO glass has been explored by using current-time curves under high-field anodization. Furthermore, we find a friendly and simple method to remove the barrier layer.

## Methods

### Deposition of aluminum thin films

Thin films of aluminum on tin-doped indium oxide (ITO) glass were formed via radio frequency (rf) sputtering process. After, that AAO layer was fabricated via anodization of the rf-sputtered aluminum films. The transparent substrate of ITO glass has a sheet resistance <7*Ω*/□. Before magnetron sputtering, the ITO glass were degreased in acetone and alcohol, and then washed in deionized water. The substrates were first vacuumed to 4×10^−5^ Pa and then inlet argon gas to the pressure of 2.2×10^−2^ Torr, the highly pure aluminum (99.99%) was deposited with the power of 200 W at room temperature. The mainly sputtering process was sputtered in one step for 1 h, as a contrast, the rest was sputtered in two steps, each step for 30 min.

### Anodization process

After deposition, the glass was cut to the dimensions of 1×1 cm^2^. Then, the samples were put into a Teflon holder with a certain contact surface exposed to the electrolyte solution. All anodization processes were carried out in an electrochemical cell equipped with a cooling system. At the same time, a DC digital controlled stirrer with a stirring rate of 400 rpm was employed to keep the temperature stable.

For the samples anodized at target voltages of 195 V, the electrolyte was the mixture of ethanol and water with a ratio of 1:4 in volume, in which the concentrations of phosphoric acid were 2.5 wt.% and the temperature was −4°C; the sample was anodized for an ultrashort time (30 to 150 s). To enlarge the holes, a phosphoric solution with the concentrations of 5 wt.% was employed at 45°C, with the time of 20 min and 30 min.

As for the rest of the samples, the target voltage was 40 V and the anodization process was performed in an electrolyte of water in which the concentrations of oxalic acid were 0.3 M. The temperature was 4°C, and the anodizing time range was 15 to 105 min.

### Characterization

The current-time transients of the anodization were record by a programmed power source (Agilent, N5752, Santa Clara, CA, USA) linked to a computer. Field emission scanning electron microscopy (FESEM) micrographs were obtained by FE-SEM Philips Sirion 200 (Amsterdam, The Netherlands) to analyse the structure of the AAO films.

## Results and discussion

### Fast anodization process in phosphoric acid

**Figure 1:**
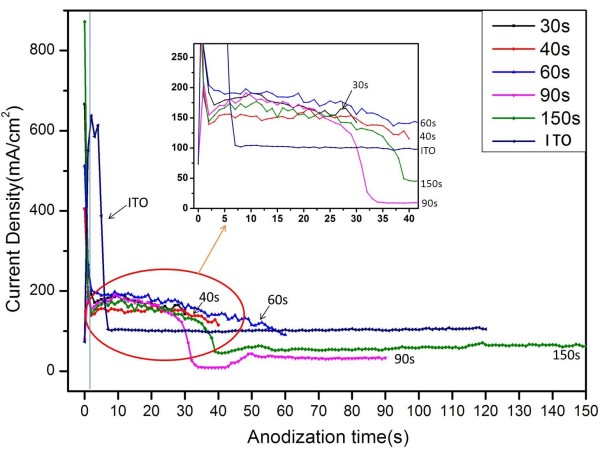
**Current-time curves of high-field anodization of bare ITO glass and sputtered aluminum.** Bare ITO glass (120 s) and sputtered aluminum for different times (30, 40, 60, 90, and 150 s).

**Figure 2:**
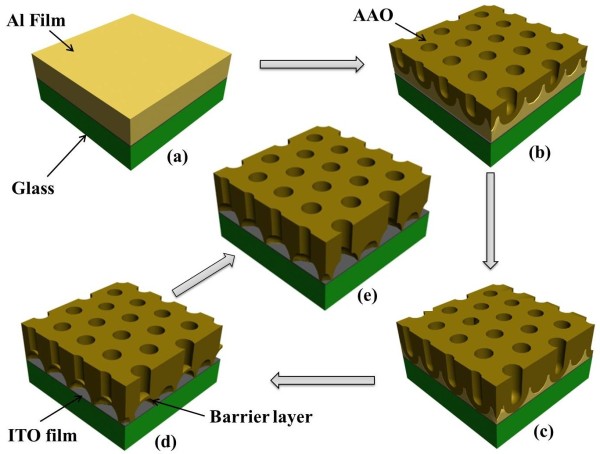
**Schematic diagram illustrating the pore formation mechanism in anodic alumina.(a)** Original aluminum sputtered on ITO glass; **(b)** the pore progress through the aluminum film and their tending towards an ordered hexagonal arrangement; **(c)** barrier layer reaching the substrate, **(d)** fully formed AAO film with barrier layer; and **(e)** the disappearance of the barrier layer.

Raising the current density, the AAO film can be formed efficiently, as shown in Figure 1, in which curves of the current density were recorded during the anodization of bare ITO and thin Al films (2 µm) in 5 wt.% phosphoric acid solution at 195 V. The anodic current density of bare ITO glass surged first, and after the initial stage, it decreased rapidly to a steady value of 100 mA/cm^2^. Other lines are the anodization curves of the sputtered aluminum with the anodizing time of 30, 40, 60, 90, and 150 s. Apparently, the anodization curves of these sputtered aluminum has a similar process, indicating that the process has an excellent repetition. At the first stage, which happens at 0 to 2 s, the curves show a dramatic decrease in current density. As Hill et al. have reported [21], this is owing to the formation of planar surface oxide on the aluminum film, and the resistance of the electrode increases as the surface oxide layer continues to grow. The second stage happens at 2 to 6 s [27], when the oxide changed to a dimpled array under the force of interfacial electric field. In this stage (2 to 6 s), in spite of the change in surface morphology, the surface oxide thickness at the bottom of the pores remains relatively constant. The oxygen through the oxide layer can be driven by the electric field as before, so the electrochemical oxidation of aluminum continues. When the surface layer had dimples, electrochemical reaction occurs at these dimple sites preferentially. With the dimples continuing to bore into the aluminum and grow into fully formed pores, the active surface area increases substantially. This increase in electrode surface area leads to the increase in current density since it is relative to the initial planar electrode surface area. Shortly after this process, the continued growth of the pores does not cause any increase in active electrode surface, so is the next stage (6 to 30 s). During this stage, just as Guo et al. observed [28], the pores grow toward the substrate and the current remains constant, which is similar to the pore growth stage in Al foils since there is no change in electrode surface area. And then comes the next stage (30 to 40 s), as anodization is about to be completed, the current falls off rapidly. This drop is due to the increase in sheet resistance owing to the diminishing amount of aluminum remaining in the film. The remaining aluminum is oxidized, leaving behind a barrier layer. And finally, in the last stage (after 40 s), the current density increases slowly and slightly and then is kept to a fixed value. However, the fixed value is lower than the current density of bare ITO, indicating that AAO is stuck to the ITO substrate. The tiny increase in this stage may be due to the upturned and broken barrier layer, as shown in Figure 2c,d, in which the holes open up a little and the exposed area of the ITO substrate to the electrolyte is increased. Chemical dissolution and field-assisted dissolution of the barrier layer can also happen during this stage; however, the process is too slow to be observed.

**Figure 3:**
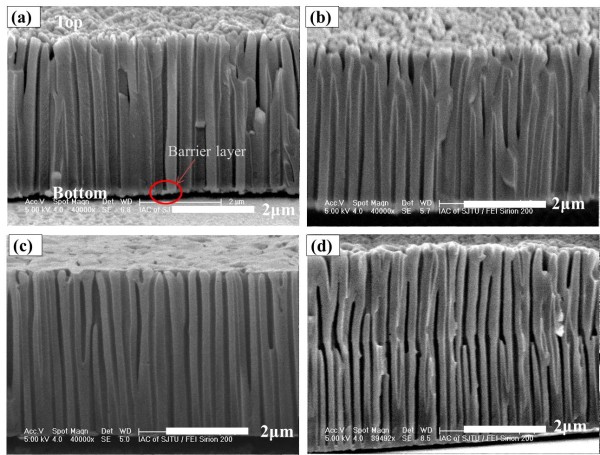
**Cross-sectional images of sample and high-field anodic alumina films with different anodizing times.** High-field anodic alumina films: **(a)***t* = 30 s, **(b)***t* = 90 s, and **(c)***t* = 150 s. Sample: **(d)***t* = 40; this sample is sputtered in two steps.

Figure 3 shows SEM images of the cross-sectional morphology of the AAO films formed from the anodization of aluminum samples at 195 V for different times. Barrier layers could be observed clearly in each image. Figure 3a,b,c has anodizing times of 30, 90, and 150 s, separately, and from these three images, we can see that the barrier layer became thinner with the increase of anodization time. This may be generated by the chemical dissolution and field-assisted dissolution of the barrier layer. The same phenomenon has been observed in the AAO walls. Figure 3d shows the anodization of the specimen sputtered in two steps and the ‘Y’ branches are obtained in the middle of the AAO walls. It can be seen clearly that the pores from the underlayer are denser than that of the upper layer. It is obvious that this phenomenon is quite different from the above three specimens whose aluminum were sputtered only in one step and shows that the ‘Y’ branches could only be developed from specimens sputtered in more than one step under high current density. Moreover, as observed by Chu et al. [23], the samples sputtered in multi-cycles and anodized under 130 V have transverse holes, which is also quite different from what we have observed in our study. The difference in morphology may be caused by the increase in anodization voltage. For the growth of the AAO film, we face a different situation when we reach the interface of the two-step sputtering process. There are defects and little voids at the interface layer. Owing to the high current density, a new growth point is formed and new branches stretch out. As a result, ‘Y’ branches appear in the middle of the specimens.

**Figure 4:**
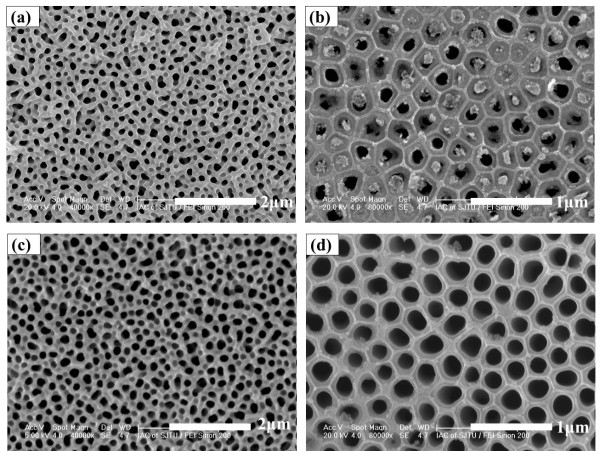
**SEM images of AAO films anodized in high field after pore widening.** Pore widening for 20 min: **(a)** top and **(b)** bottom views. Pore widening for 30 min: **(c)** top and **(d)** bottom views.

Figure 4 shows the top and bottom views of AAO after the pore widening process. In this process, a further attempt to broaden the range of pore diameters and lengths was obtained for AAO films on ITO. The FESEM images of Figure 4a,b show the aluminum films anodized in phosphoric acid and pore widening for 20 min. And the FESEM images of Figure 4c,d show the aluminum films anodized in phosphoric acid and pore widening for 30 min. Figure 4a,c shows top views, while Figure 4b,d shows bottom views. All samples showed randomly distributed nanopores with irregular shapes and sizes. After pore widening, the pores can be observed more clearly. The pores in Figure 4a are smaller than those in Figure 4c. A barrier layer still exists in Figure 4b, while in Figure 4d, the barrier layer has been removed. This illustrates that as pore widening time increases, the pores are enlarged and opened.

### Anodization in oxalic acid

**Figure 5:**
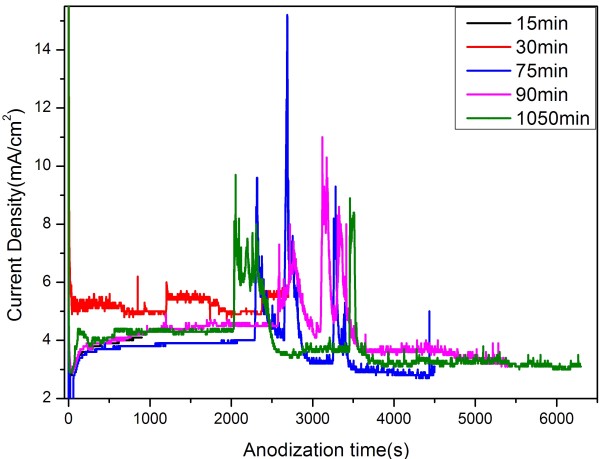
Current-time curves of low-field anodization of sputtered aluminum for different times (15, 30, 75, 90, 105 min).

**Figure 6:**
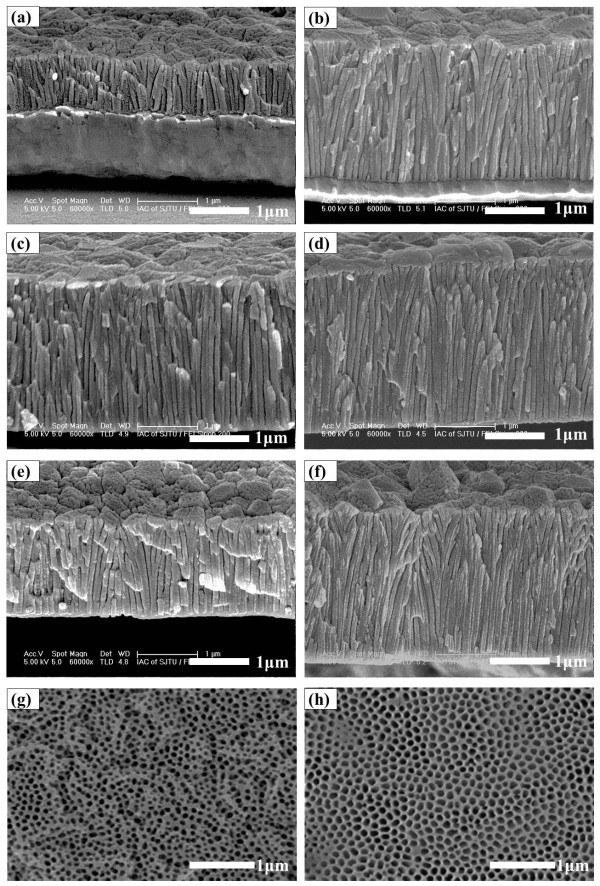
**Cross-sectional images and top and bottom views of AAO and cross-sectional image of Al.** AAO is anodized in oxalic acid for different times: **(a)** 15, **(b)** 30, **(c)** 75, **(d)** 90, and **(e)** 105 min. **(f)** Al sputtered in two steps anodized for 75 min. AAO afer pore widening: **(g)** top and **(h)** bottom views.

Current density as a function of anodizing time is shown in Figure 5. The five curves are specimens anodized for different times and the specimens are Al sputtered on ITO glass for 1 h in one step and all the five curves share the same characteristics. It decreased rapidly first and then rose to the value ca. 4 mA/cm^2^. After keeping to this value for a long time, the current density had swings. Finally, the current densities drop to a fixed value of about 3 mA/cm^2^, till the process ended. The process before 2,000 s can be explained as Figure 1. It is the swings that makes it different from the former process. These swings generated when the barrier layer reach the bottom of Al and touch the glass, which can be determined from cross-sectional images shown in Figure 6. As the top of the barrier layer reached the ITO glass substrate, the continuous Al film transformed into the Al pyramids between the pores. Different from the conditions of the high electric field, the low electric field would demand much more time in consuming the remaining Al pyramids. Therefore, there would be some inhomogeneity regions since the initial surface of Al was uneven. When the barrier layer in some regions opened up, the current density surged. The increase of this value appears distinct owing to the low value of the overall current density. The ITO layers in some parts of this region were broken then and the current density reduced. This is the reason why the swings were generated. After the fluctuation period, current densities decreased and maintained to the value of about 3 mA/cm^2^, which is lower than the initial fixed value of about 4 mA/cm^2^. This is also similar to the curves in Figure 1.

**Figure 7:**
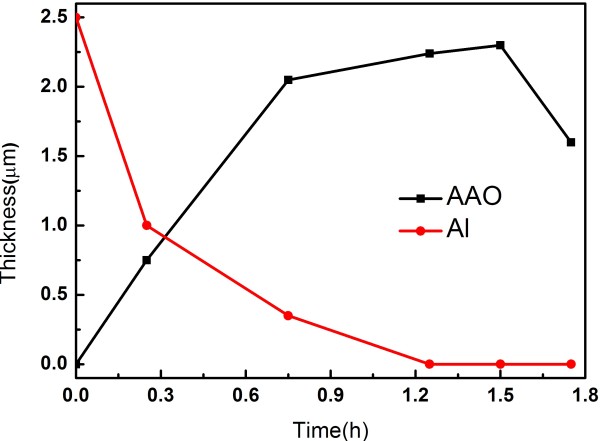
**Changes of film thickness with anodizing time.** The red line is the change in aluminum thickness and black line is the change in porous alumina thickness.

Figure 6 is the FESEM images anodized in oxalic acid for different times. The thickness of AAO films increased and the thickness of aluminum layers decreased with the anodization process going on. Figure 6a is the specimen anodized for 15 min, in which the thickness of Al is equal to the thickness of AAO. The specimen in Figure 6b is anodized for 30 min with the AAO almost formed and a thin Al layer remaining. However, the specimen in Figure 6c has very few Al and the anodizing time reaches 75 min. In Figure 6d, whose anodizing time reaches 90 min, the AAO layer gets even thicker and the barrier layer is upturned. What is interesting is that as the time reaches 105 min, the AAO layer gets thinner and there are some tips without barrier layers, which is shown in Figure 6e. What is more, in this kind of process, is that ‘Y’ branches would not appear with specimens sputtered in two steps, as shown in Figure 6f. There may be two reasons for this phenomenon. One reason is that, with slower anodization, the AAO films become more compact. The other reason may be that the acidity of phosphoric acid is stronger than oxalic acid. Irregular shapes and sizes are randomly distributed in Figure 6g,h, which are the top and bottom views of AAO anodized in oxalic acid after pore widening process. The change of thickness can be seen clearly from Figure 7. The red line is the thickness curve of AAO and the black line is that of Al. It can be seen clearly that the AAO layer got thicker at first and then decreased while the Al layer gets thinner with the progress of anodization.

Figure 2 is the anodizing schematic of the former process. Figure 2a shows Al film sputtered on ITO glass. When immerged in electrolyte, the AAO layer is formed, as shown in Figure 2b. After anodizing for a long time, the barrier layer touches the bottom, reaching the ITO glass which can be seen in Figure 2c. As the anodizing time goes on, the barrier layer upturned and there is no aluminum left as Figure 2d shows. And the remaining barrier layer can be removed as this process goes on, leaving an AAO template without barrier layer, as shown in Figure 2e.

**Figure 8:**
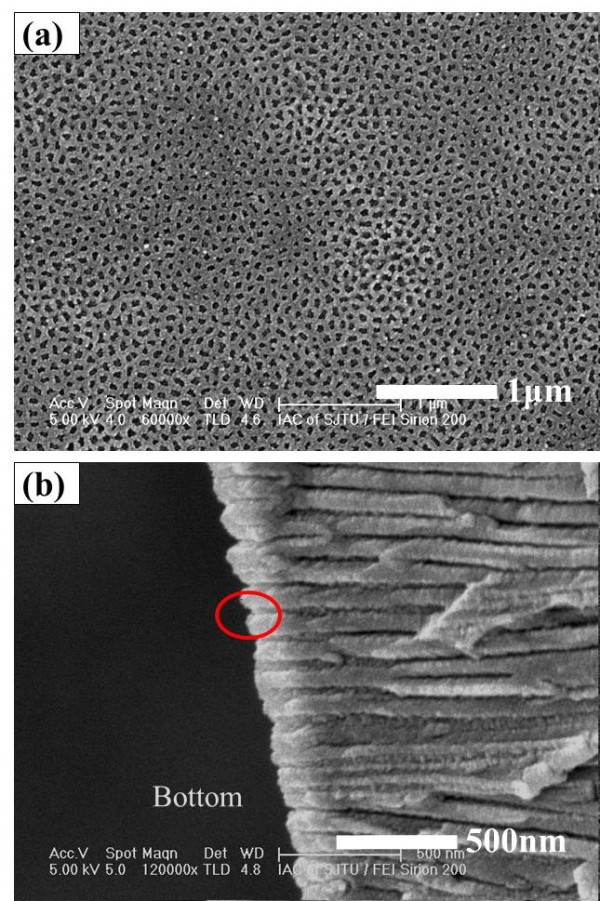
**SEM image of AAO without barrier layer by anodizing in oxalic acid at 40 V for 2 h.(a)** bottom view, **(b)** cross-sectional view.

Figure 8 shows the bottom of AAO anodized in oxalic acid at 40 V for 2 h, twice the time for the Al layer to run out as shown in Figure 6c. These images indicate that the barrier layers are totally opened. Figure 8a is the bottom view of AAO. In this image, we can see that there are no barrier layers left in the template. The holes are distributed randomly. Figure 8b is the cross-sectional image of AAO; the side view of the bottom can be seen and the bottom is apparently open. This phenomenon can provide a powerful evidence that the barrier layer can be removed as shown in Figure 2e.

## Conclusion

In this study, an efficient way to form AAO film on ITO glass is performed, reducing the anodizing time to about 30 s. The forming process of AAO on ITO has been explained based on the current-time curves. The thickness of the AAO film anodized in oxalic acid increased first and then decreased with the progress of the anodization process. Getting rid of barrier layer has been proved to be the key to make electrical contact at the bottom, which helps to assemble nanowire structures on ITO glass directly. Having enough anodizating time, the barrier layer could be eliminated. This method will be highly advantageous to form nanostructured photoelectric devices.

## Competing interests

The authors declare that they have no competing interests.

## Authors’ contributions

SDL participated in the design of the study, carried out the experiments, and performed the statistical analysis, as well as drafted the manuscript. ZZX and CQZ helped in the experiments and data analysis. LM participated in the design of the experimental section and offered help in the experiments. MJZ participated in the design of the study, provided the theoretical and experimental guidance, performed the statistical analysis, and revised the manuscript. WZS gave his help in using the experimental apparatus. All authors read and approved the final manuscript.
